# Grandparentage assignments identify unexpected adfluvial life history tactic contributing offspring to a reintroduced population

**DOI:** 10.1002/ece3.2378

**Published:** 2016-08-26

**Authors:** Nicholas M. Sard, Dave P. Jacobson, Michael A. Banks

**Affiliations:** ^1^ Department of Fisheries and Wildlife Coastal Oregon Marine Experiment Station Hatfield Marine Science Center Oregon State University 2030 SE Marine Science Drive Newport Oregon 97365

**Keywords:** Adfluvial salmon, grandparentage assignment, life history tactics, reintroduction

## Abstract

Diversity in life history tactics contributes to the persistence of a population because it helps to protect against stochastic environments by varying individuals in space and time. However, some life history tactics may not be accounted for when assessing the demographic viability of a population. One important factor in demographic viability assessments is cohort replacement rate (CRR), which is defined as the number of future adults produced by an adult. We assessed if precocial resident males (<age‐3) and adfluvial Chinook salmon (*Oncorhynchus tshawytscha*), adults that reside in freshwater their entire lives, contributed offspring to a reintroduced population from 2008 to 2013. We found that 9 ± 5% of offspring with an unassigned parent remained unexplained after accounting for sources of human error. Using grandparentage assignments, we identified 31 precocial resident males and 48 probable adfluvial Chinook salmon produced by anadromous mate pairs from 2007 to 2012. Previously published CRR estimates for the 2007 and 2008 reintroduced adults, based on only anadromous returning adult offspring, were 0.40 and 0.31, respectively. By incorporating adfluvial females, we found CRR estimates increased by 17% (CRR: 0.46) and 13% (CRR: 0.35) for the 2007 and 2008 cohorts, respectively.

## Introduction

Diverse portfolios in both economics and ecosystems help to increase “additive returns” in stochastic environments (Figge 2004; Koellner and Schmitz 2006). Similarly, many plants and animals increase the chance of population persistence (Boer 1968; Fox 2005) through life history tactics that vary individuals in space and time (e.g., dormancy and dispersal, Weaver et al. 1996; Rubio de Casas et al. 2015). Life history diversity may help populations established through reintroduction persist into the future (e.g., Greene et al. 2009); however, this topic has received little attention in the literature. Variation in anadromy among individuals within salmonid populations (e.g., precocial resident males, partial migration, and freshwater lake populations, Ricker 1938; Taylor 1989; Rohde et al. 2014) offer an excellent opportunity to study how life history diversity contributes to the persistence of reintroduced populations because there are several reintroductions currently ongoing throughout the Pacific Northwestern United States (Anderson et al. 2014).

The persistence of a reintroduced population, in part, rests on its demographic and evolutionary viability through time (Anderson et al. 2014; Robert et al. 2015). One important factor when evaluating the demographic viability of a population is cohort replace rate (CRR), defined as the number of future adults produced by an adult (Botsford and Brittnacher 1998), because estimates help determine whether a population will persist into the future after controlling for immigration. Understanding how variation in life history tactics contribute to a reintroduced population can improve estimates of CRR by accounting for all forms of sexually mature adults.

Grandparentage assignment methods have recently arisen as a way to identify unsampled adults contributing to a population (Letcher and King 2001). To our knowledge, this approach has only been applied to salmonid populations; however, grandparentage assignments could be applied to any taxa that have genetic pedigrees assembled over multiple generations. Grandparentage assignment methods take advantage of the fact that grandparent pairs share one in four of their alleles at a given locus with their grandchildren, just as each diploid parent shares one of its two alleles with its offspring. Extending the exclusionary concept in parentage assignments to grandparent pairs has brought insights into gene flow between resident and anadromous life histories (Christie et al. 2011), and the spawning success of resident hatchery origin Chinook salmon (*Oncorhynchus tshawytscha*) (Ford et al. 2015).

Spring Chinook salmon from the upper Willamette River basin are an Evolutionary Significant Unit (ESU) (NMFS, 2005, 2008) listed as threatened under the U.S. Endangered Species Act (ESA). Oregon Department of Fish and Wildlife and the National Marine Fisheries Service have developed a recovery plan for Chinook salmon in the Willamette River basin (ODFW and NMFS, 2010). As part of this plan, anadromous Chinook salmon adults are being reintroduced above several dams in the Willamette River basin to provide access to historical spawning habitat. In the case of the Cougar Dam system, adults have been collected at a nearby hatchery and reintroduced to historical habitat above the dam via fish transportation trucks annually since 1996 (Zymonas et al. 2010). Along with hatchery origin fish, natural origin Chinook salmon adults have also been collected at a trap and haul facility built at the base of Cougar Dam and reintroduced above the dam since 2010. This trap and transport reintroduction approach has enabled the near complete tissue sampling of all the potential anadromous adult parents, thereby allowing for estimates of CRR. Contrary to recently published studies for other Chinook and coho (*O. kisutch*) salmon reintroductions (Anderson et al. 2015; Evans et al. 2015), current CRR estimates suggest that the population above Cougar Dam is not replacing itself (Sard et al. 2016).

The ability to have a more complete understanding of CRRs for reintroduced populations is limited by excluding difficult to sample, or unanticipated, life history tactics from estimates. Sard et al. (2016) used genetic parentage assignments to estimate CRR for the population established above Cougar Dam by the reintroduction program. This approach identified anadromous adult offspring returning in subsequent years; however, it does not account for any adults that sexually matured above Cougar Dam because these individuals were never encountered and therefore were not sampled.

There are two types of unsampled sexually mature adult Chinook salmon possible above Cougar Dam. First, some precocial resident males become sexually mature in freshwater rivers at age 1 or age 2 and avoid ocean migration altogether (Taylor 1989). Second, as an alternate strategy, some male and female salmon may become sexually mature, presumably in the freshwater reservoir, and migrate upstream to spawn as adults older than age 2, hereafter adfluvial Chinook salmon. There are few studies regarding adfluvial Chinook salmon in the literature. However, this life history tactic has been recently identified in another reservoir–river system in the Willamette River basin (Romer and Monzyk 2014). Perales et al. (2015) provided additional evidence that adfluvial Chinook salmon can survive and reproduce in several reservoir–river systems in California, USA. These studies suggest that the existence of adfluvial Chinook salmon may be more common than once thought.

The Chinook salmon reintroduction above Cougar Dam has also been intensively sampled for age‐0 juveniles produced and collected above the dam, which has informed managers of factors related to the spawning success of reintroduced adults (Sard et al. 2015). Given this study system, one expectation is that most age‐0 juveniles should assign to both parents because the offspring were collected above the dam and nearly all anadromous adults that could have produced them were sampled (see Sample collection below). However, as reported in Sard et al. (2015), among four genetic pedigrees, only 79 ± 7% (mean ± SD) of juveniles assigned to both parents reintroduced from 2008 to 2011. There are a few explanations for this observation, and therefore it is important to test multiple working hypotheses to avoid false or biased conclusions (Chamberlin 1965).

Four hypotheses that may explain offspring with unassigned parents in each adult–juvenile genetic pedigree include the following: (1) genotyping error; (2) some anadromous adults lacked genotypes because of missing tissue samples or poor DNA quality; (3) incorrect sex identification of some anadromous adults that resulted in unintentional exclusion of a parent based on the Bayesian likelihood approach taken; and (4) the existence of unsampled parents residing above Cougar Dam. Here, we test whether life history diversity as expressed by resident precocial males and/or adfluvial Chinook salmon (Hypothesis 4) contributed offspring to the reintroduced Chinook salmon population above Cougar Dam after accounting for sources of human error (Hypotheses 1–3). Our findings suggest that precocial resident males and adfluvial Chinook salmon indeed reside above the dam. We provide information on the inferred ages of sexually mature male and female adfluvial Chinook salmon, the number spawning each year, as well as their spawning success with anadromous mates.

## Methods

### Sample collection

**Figure 1:**
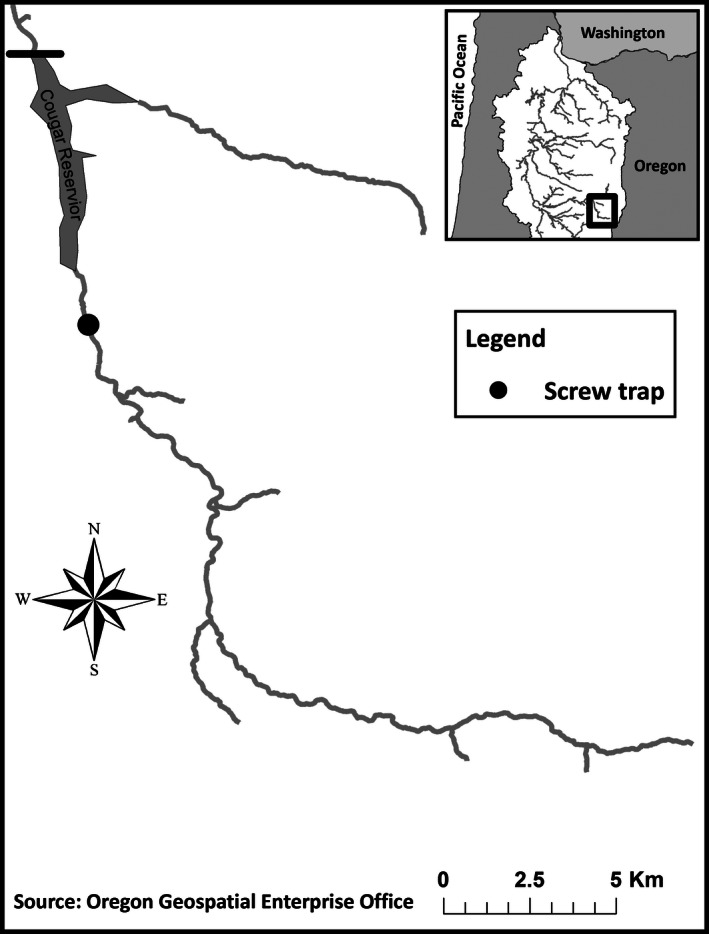
Cougar Dam, indicated by the solid black horizontal line, is located on the South Fork of the McKenzie River. Juveniles were collected in a screw trap located above the dam.

Since 1996, hatchery origin adult Chinook salmon have been reintroduced annually above the 158 m tall Cougar Dam, which is located on the South Fork of the McKenzie River, Oregon, USA (Fig. 1). Between 2007 and 2013, nearly all anadromous adults (99%, *n* = 6115/6119) reintroduced above the dam have been tissue‐sampled for subsequent DNA extraction (Table 1). The number of anadromous adults reintroduced above Cougar Dam each year ranged from 687 to 1386 (Table 1). In addition, tissue was collected from a subsample of age‐0 juvenile Chinook salmon captured in a screw trap above the dam from 2009 to 2014, of which 2160 ± 335 were sampled for genetic analysis annually (Table S1). Each year, the number of juveniles sampled for genetic analysis was proportional to the total number sampled in the screw trap each month (Sard et al. 2015). We isolated DNA from adult and juvenile fin clips using a protocol developed by Ivanova et al. (2006). Samples were genotyped at 11 microsatellite loci: *Ots201*,* Ots208b*,* Ots209*,* Ots211*,* Ots212*,* Ots215*,* Ots249*,* Ots253*,* Ots311*,* Ots409*, and *Ots515* (Banks et al. 1999; Naish and Park 2002; Williamson et al. 2002; Greig et al. 2003). Polymerase chain reaction products were visualized on an ABI 3730XL DNA analyzer and size scored against Liz500 using GeneMapper software (Thermo Fisher Scientific, Waltham, MA). Genotyping error rate was estimated by randomly sampling 1% of genotyped individuals and re‐genotyping them at the 11 microsatellite loci described above. Genotyping error rate was calculated by dividing the number of discordant alleles scored by the total number of alleles scored. We calculated genotyping error rate for each genetic pedigree separately so that estimates could be used in simulations (see Hypothesis 1: Genotyping error, Table S2).

**Table 1:** Summary of the number of anadromous adults reintroduced above the dam, number of missing tissue samples, and the number of individuals missing more than one genotype are described for each sex from 2007 to 2013

Year	Sex	*N*	Missing	
			Tissue	>1 GT
2007	Female	318	0	0
	Male	428	0	3
2008	Female	288	0	0
	Male	585	0	0
2009	Female	604	0	3
	Male	782	1	10
2010	Female	264	0	4
	Male	484	1^a^	6
2011	Female	324	1^a^	5
	Male	407	0	1
2012	Female	439	0	1
	Male	509	0	1
2013	Female	337	0	2
	Male	350	1^a^	0

a Adults were randomly assigned a sex.

Details regarding the genetic parentage assignment of reintroduced adults to returning anadromous adult offspring, as well as age‐0 offspring collected above the dam have previously been published (See Sard et al. 2015, 2016). In short, adults were assigned to returning adult offspring (adult‐adult genetic pedigree) using both CERVUS (Marshall et al. 1998; Kalinowski et al. 2007) and COLONY (Jones and Wang 2010). Anadromous adults were assigned to age‐0 juveniles (adult‐juvenile genetic pedigrees) using SOLOMON (Christie et al. 2013). All parent–offspring pairs were allowed to mismatch at no more than one locus. Among the 11 loci used, a high number of alleles (A = 34 ± 1) and high heterozygosity (*H*_o_ = 0.92 ± 0.01) were observed annually, which in turn resulted in low nonexclusionary probabilities (<1.85E^−07^) and low expected numbers of false parent–offspring (<5) assignments (see Sard et al. 2015, 2016). Collectively, there was sufficient power to correctly resolve parent–offspring relationships, if present.

Methods described in Sard et al. (2015) were used to assemble two additional adult–juvenile genetic pedigrees with the 2012 and 2013 reintroduced anadromous adults and samples of age‐0 offspring collected above the dam in 2013 (*n* = 2792) and 2014 (*n* = 2087), respectively (Table 1, Table S1). For each of the six adult–juvenile genetic pedigrees, the percent of juveniles with no parent assigned, unassigned mothers, unassigned fathers, and both parents assigned was calculated. We predicted that more juveniles would have unassigned fathers compared to unassigned mothers if only precocial resident male Chinook salmon (< age‐3) were present above Cougar Dam. Therefore, we tested whether the median percent of offspring with unassigned mothers differed compared to those with unassigned fathers using a Wilcoxon rank‐sum test.

Throughout this manuscript, we focus the analysis on juvenile offspring with one unassigned parent. This approach enabled us to compare results from the genotyping error, missing anadromous adult genotypes, and incorrect sex identification of anadromous adults hypotheses with the fourth hypothesis, missing unsampled parents above the dam, which were identified by grandparentage assignments with one parent known (see Hypothesis 4: Unsampled adults above the dam and Christie et al. 2011).

### Hypothesis 1: Genotyping error

The percent of juveniles with one unassigned parent due to genotyping error was estimated using simulations. First, SOLOMON (Christie et al. 2013) was used to simulate microsatellite genotypes for parents and offspring using allele frequencies estimated from the study population. The number of parents used, as well as the genotyping error rate used for each simulation were specific to each adult‐juvenile pedigree assembled, since each parameter varied annually (Table 1, Table S2). SOLOMON uses a uniform reproductive success distribution, which prevented simulating the exact number of offspring used in each adult–juvenile genetic pedigree. We chose a number of offspring produced by each parent that resulted in a simulated number of offspring genotypes as close as possible to that used in each of the actual genetic pedigree (Table S1). However, the choice of the number of offspring produced by each parent did not substantially change estimates for the percent of offspring with one unassigned parent explained by genotyping error (unpublished data). Simulated parents were assigned to offspring with SOLOMON's no‐known parent Bayesian likelihood approach using methods described in Sard et al. (2015). For each simulated genetic pedigree, the expected percent of offspring missing one parent due to genotyping error alone was calculated. We tested whether the observed median percent of juveniles with one unassigned parent differed from that expected by genotyping error using a Wilcoxon rank‐sum test to determine whether Hypothesis 1 alone could explain why offspring had one unassigned parent.

### Hypothesis 2: Missing anadromous parents

The same approach in Hypothesis 1 was used to test Hypothesis 2, except that a number of known simulated adult samples were randomly removed before making parent–offspring assignments and assembling each simulated adult–juvenile genetic pedigree instead of incorporating genotyping error. The numbers of females (2 ± 2) and males (3 ± 4) removed were based on the number of known anadromous adults missing a genotype at more than one locus, as well as the number of adults missing a tissue sample (Table 1). The sex for three Chinook salmon was randomly assigned because we were unable to genotype them at the sex linked marker *Oty3* (Brunelli et al. 2008; Table 1). Following each of the six simulations, one for each adult–juvenile genetic pedigree, the expected percent of juveniles with one unassigned parent due to missing anadromous parent genotypes was calculated each year. We tested whether the observed median percent of juveniles with one unassigned parent differed to that expected by missing anadromous parent genotypes using a Wilcoxon rank‐sum test to determine whether Hypothesis 2 alone could explain why offspring had one unassigned parent.

### Hypothesis 3: Incorrect sex identification

We tested whether some adults were incorrectly sexed using the exclusion one‐parent known option in SOLOMON. All offspring with one parent assigned were evaluated as a known parent–offspring pair. These parent–offspring pairs were assigned to all adults that were the same sex as the known parent. Only adults that did not assign to other juveniles in the genetic pedigree that year were used for two reasons. First, there was strong evidence that most of the sex designations we have made were correct because both Banks et al. (2014) and Brunelli et al. (2008) found high concordance between genotypic and phenotypic sex in this system (90 and 100%, respectively). It is possible that both genotyping error and the lack of secondary sexual phenotypes among early returning adults caused some of the discordance observed in Banks et al. (2014). Regardless, adults assigned to one or more offspring likely had the correct sex designation. Secondly, we wanted to limit the number of pairwise comparisons made, thereby limiting the chance of false assignment. The one‐parent known option in SOLOMON requires that all parent–offspring must match at all loci and there must be no missing data. As a result, 32 ± 25 parent–offspring assignments were not included in this analysis (Table 2). Assignments that matched at all loci were considered as evidence that the sex of the assigned parent was incorrect. The percent of offspring explained by incorrect sex identification was calculated by dividing the number of assignments made by the total number of offspring used in each adult–juvenile pedigree. We tested whether the observed median percent of juveniles with one unassigned parent differed to that expected by incorrect sex identification using a Wilcoxon rank‐sum test to determine whether Hypothesis 3 alone could explain why offspring had one unassigned parent.

**Table 2:** Number of parent–offspring pairs included/excluded for offspring only assigned to a father or mother for each adult–juvenile genetic pedigree, as well as the number of assignments explained by incorrect sex identification from 2008 to 2013

Year	Included		Excluded		Assignments	
	Father only	Mother only	Father only	Mother only	Father only	Mother only
2008	117	134	0	0	2	0
2009	193	109	88	61	5	0
2010	319	57	27	17	18	0
2011	319	188	49	29	1	3
2012	301	131	26	44	7	0
2013	71	57	16	24	5	0

### Hypothesis 4: Unsampled parents above the dam

We identified 218 (55 ± 80, annually) and 2616 (435 ± 145, annually) unique mate pairs present in the adult–adult and adult–juvenile genetic pedigrees, respectively (Table S3). These mate pairs were used as grandparent pairs because there was strong genetic evidence that these individuals mated in the wild based on low non‐exclusionary parent pair (9.8 × 10^−17^± 3.9 × 10^−17^) probabilities (see Sard et al. 2015, 2016). This approach reduced the chance of false‐positive grandparent pair‐grandoffspring assignments by limiting pairwise comparisons to only grandparent pairs for which there was evidence for their existence. The expected number of false grandparent–grandoffspring trios was calculated using the genotypes of grandparent pairs and potential grandoffspring with either an unassigned mother or father in each of our six adult–juvenile genetic pedigrees (Christie et al. 2011). This analysis focused on grandoffspring that had one parent assigned because it reduced false‐positive assignments by excluding alleles in the offspring explained by the assigned anadromous parent (Christie et al. 2011). Initially grandparent pair‐grandoffspring trios (Gtrios) that matched at all 11 loci were accepted. This hypothesis was further tested by genotyping all putative Gtrios identified at an additional four loci: *Ogo2, Ogo4, Ssa408, OtsG474* (Olsen et al. 1998; Cairney et al. 2000; Williamson et al. 2002). We accepted Gtrios that matched at all 15 loci for further analyses. We tested whether the observed median percent of juveniles with one unassigned parent differed from that explained by unsampled Chinook salmon identified by grandparentage assignments using a Wilcoxon rank‐sum test to determine whether Hypothesis 4 alone could explain why offspring had one unassigned parent.

We conservatively assumed each unique grandparent pair that assigned to at least one grandoffspring only produced one unsampled adult. This assumption enabled estimates of the number of unsampled adults produced by anadromous mate pairs that contributed offspring to the population each year, as well as the number of unsampled male and females that were successful at spawning from age 1 to age 6. We tested for differences in age at reproduction among the unsampled males and females identified by grandparentage assignments using a Wilcoxon rank‐sum test. Any unsampled females identified using grandparent pairs that mated in 2007 or 2008 were incorporated into female CRR estimates for each cohort.

All analyses were conducted in R version 3.1.1 (R Core Team, 2014). All *P*‐values associated with testing why offspring had one unassigned parent were false discovery rate corrected (Benjamini and Hochberg 1995).

We provide three resources for use in future grandparentage studies, which include two R scripts (modified from SOLOMON, Christie et al. 2013) to simulate (including allele frequencies used in this study) the expected number of offspring with unassigned parents due to genotyping error and missing anadromous parents, as well as an R script (created by NMS) to identify Gtrios after accounting for one parent's genotypes on GitHub (https://github.com/nicksard/grandparentage).

## Results

### Genetic pedigrees

**Figure 2:**
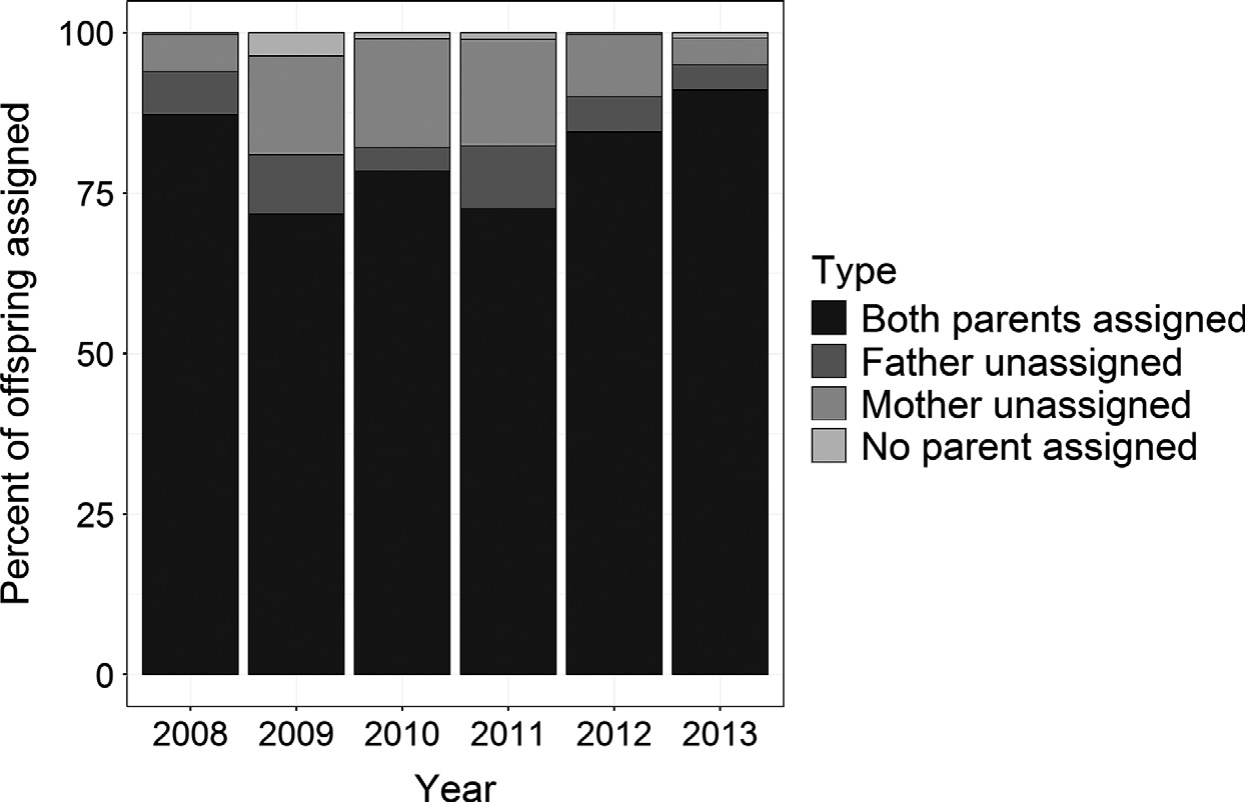
Percent of offspring that had both parents assigned, an unassigned mother or father, or no parent assigned from 2008 to 2013.

We found 15% of the offspring in the 2012–2013 adult–juvenile genetic pedigree had one unassigned parent, which was comparable to the four previously published adult–juvenile genetic pedigrees (range: 13–26%). However, the 2013–2014 genetic pedigree had the lowest percent of unassigned parents (8%, Fig. 2). Overall, 18 ± 7% of offspring had one unassigned parent among the adult–juvenile genetic pedigrees assembled. The percentage of offspring with an unassigned father (6 ± 3%) compared to those with an unassigned mother (11 ± 7%) did not differ statistically (*V* = 28, *P* = 0.132).

### Hypothesis testing

Hypotheses associated with human error could not explain all the offspring with one unassigned parent. The estimated percent of offspring with one unassigned parent due to genotyping error (7 ± 6%, Table S2), missing anadromous parent genotypes (1 ± 1%, Table S2), and incorrect sex identification (0.3 ± 0.3%, Table S2) differed significantly to that observed in adult–juvenile genetic pedigrees (*W* = 33, *P* = 0.02; *W* = 36, *P* = 0.01; *W* = 36, *P* = 0.01, respectively). Genotyping error accounted for most of the unassigned parents (39 ± 22%), whereas missing anadromous parents (5 ± 5%) and incorrect sex identification (2 ± 1%) accounted for few unassigned parents (Table S2). However, some offspring remained unexplained (9 ± 5%, Table S2) after testing Hypotheses 1–3 in aggregate (*W* = 31, *P* = 0.049).

We found genetic evidence that 2 ± 2% of the offspring with one unassigned parent were due to the existence of precocial resident males and adfluvial Chinook salmon residing above Cougar Dam (Table 3). A total of 244 grandoffspring were assigned to grandparent pairs based on the original 11 loci. After incorporating genotypes at an additional four loci (*A* = 16 ± 5 and *H*_o_ = 0.83 ± 0.07), a total of 227 Gtrios matched at all 15 loci (Table 3). The percent of Gtrios that identified adfluvial males versus females were similar after including the additional loci (41 and 59%, respectively, Table 3). Most (15/17) of the exclusions were observed because we were unable to successfully genotype at least one individual involved in the Gtrio at the additional loci (Table S4). Difficulty in amplifying the additional loci for these individuals was likely due to poor DNA quality in the tissue samples because loci could not be amplified after attempting to re‐extract DNA from the original tissue samples. In addition, there were 18 juvenile offspring that assigned to two (*n* = 16) or three (*n* = 2) grandparent pairs. These assignments were excluded from all analyses because we were unable to identify a single grandparent pair after including additional loci.

**Table 3:** Summary of the grandparent–grandoffspring assignments (Gtrios) in the adult–juvenile genetic pedigrees. The expected (Exp.) number of false Gtrios, as well as the number of observed (Obs.) that shared alleles at 11 and 15 loci are described. We also include the unsampled parent age based on the year the grandparent pair mated (GP year) and the year the one anadromous adult assigned to the age‐0 juvenile was reintroduced

Parent year	GP year	Unsampled parent age	Unassigned father			Unassigned mother		
			Exp. False Gtrios	Obs. Gtrios		Exp. False GTrios	Obs. Gtrios	
				11 loci	15 loci		11 loci	15 loci
2008	2007	1	4	1	1	4	0	0
2009	2007	2	4	3	2^a^	4	0	0
2009	2008	1	9	0	0	13	0	0
2010	2007	3	2	0	0	10	0	0
2010	2008	2	4	17	17	20	0	0
2010	2009	1	3	2	2	18	0	0
2011	2007	4	6	9	9	9	27	25^a^
2011	2008	3	12	0	0	18	0	0
2011	2009	2	11	12	9^a^^,^ b	19	0	0
2011	2010	1	7	0	0	11	0	0
2012	2007	5	4	7	6^a^	8	46	45
2012	2008	4	10	22	22	18	37	36^a^
2012	2009	3	9	0	0	17	0	0
2012	2010	2	5	8	8^b^	9	0	0
2012	2011	1	5	0	0	10	0	0
2013	2007	6	1	0	0	2	0	0
2013	2008	5	3	0	0	4	2	2
2013	2009	4	3	1	1	3	28	27^a^
2013	2010	3	2	0	0	2	0	0
2013	2011	2	1	22	15^a^	2	0	0
2013	2012	1	3	0	0	3	0	0

a Some Gtrios were excluded because we could not amplify at least on individual in the assignment at the additional loci, see Table S4.

b Four Gtrios were moved from the unassigned mother to the unassigned father category after correcting for genotyping errors, see Table S4.

No Gtrios were observed in 48% (10/21) of the comparisons made for juveniles with unassigned fathers and 76% (16/21) with unassigned mothers (Table 3). For example, not a single Gtrio assignment was identified when the 509 grandparent pairs identified in 2008 were compared to the 115 offspring with unassigned fathers in the 2009–2010 adult–juvenile genetic pedigree; this is significant because there were more than 50,000 pairwise comparisons of genotypes made. Initially, three Gtrios identified putative unassigned mothers that would have been younger than age 3. However, based on genotyping error at the sex‐linked marker *Oty3*, the Gtrios actually identified unassigned fathers (Tables 3 and S4). For comparisons with at least one Gtrio, most (69%, 11/16) had more Gtrios observed than were expected by chance (Table 3). The percent of offspring explained by the missing adfluvial adults hypothesis differed to that of the observed data after Gtrios were genotyped at all 15 loci (*W* = 36, *P* = 0.01). Percentage of offspring with one unassigned parent observed did not statistically differ from expected when all four hypotheses were combined (*W* = 30, 0.065). However, 7 ± 4% of offspring with one unassigned parent remained unexplained after accounting for all four hypotheses.

We provide evidence that 79 unsampled sexually mature Chinook salmon spawned from 2008 to 2013 (Table 4). In total, we identified 31 precocial resident males (age 1 and age 2), and 48 male and female adfluvial Chinook salmon that reproduced at age 4 or 5. Among those inferred from grandparent assignments, unsampled males (median = 2) were younger than unsampled females (median = 4, *W* = 1339, *P* < 0.001). The number of unsampled Chinook salmon adults identified in each adult–juvenile pedigree ranged from 1 to 34 annually (Table 4). The number of juveniles assigned to a single grandparent pair ranged from 1 to 27, although this variation was in part due to the number of offspring used in assignments. We found that 34 unique grandparent pairs in 2007 produced at least one unsampled parent above the dam; whereas in 2008, 25 unique grandparent pairs were observed (Table 4). The inclusion of the adfluvial female Chinook salmon increased the CRR in 2007 from 0.40 to 0.46 (17%). Similarly, the CRR estimate for 2008 (0.31) increased by 13% (CRR = 0.35).

**Table 4:** Summary of the number of unsampled Chinook salmon by age and sex identified by grandparentage assignments

Year produced	Sex	Age						Total
		1	2	3	4	5	6	
2007	Female	0	0	0	8	13	0	21
	Male	1	2	0	7	3	0	13
2008	Female	0	0	0	11	1	–	12
	Male	0	10	0	3	0	–	13
2009	Female	0	0	0	1	–	–	1
	Male	1	4	0	1	–	–	6
2010	Female	0	0	0	–	–	–	0
	Male	0	4	0	–	–	–	4
2011	Female	0	0	–	–	–	–	0
	Male	0	9	–	–	–	–	9
2012	Female	0	–	–	–	–	–	0
	Male	0	–	–	–	–	–	0

## Discussion

We tested four hypotheses to explain unassigned parents in genetic pedigrees using a combination of parentage assignment, grandparentage assignment, and simulations. This approach indicated that some unassigned parents remained unexplained after accounting for sources of human error. As a final hypothesis, we tested for the existence of precocial resident males and adfluvial male and female Chinook salmon. We provide genetic evidence that unsampled adults resided above Cougar Dam and contribute offspring to the population. The probable existence of these adfluvial adults was corroborated by genotyping Gtrios at an additional four loci (15 total), and in some cases, unsampled parents were supported by several offspring assignments (up to 27). The incorporation of nonanadromous life history tactics appears to marginally increased CRR estimates; however, overall our study contributes to the growing body of evidence that adfluvial male and female Chinook salmon can survive above dams for their entire lives. We provide insight into how many adfluvial adults are actively spawning each year and at what age, as well as their spawning success with anadromous mates. In addition, given our data and other recently published data from Romer and Monzyk (2014) and Perales et al. (2015), it is possible that adfluvial Chinook salmon may contribute to other Chinook salmon reintroduction programs.

Salmon fisheries established in the Great Lakes in the 1900s demonstrate that male and female Chinook salmon can survive in freshwater environments for their entire lives (Emery 1985). However, the expression of adfluvial Chinook salmon life history in smaller reservoirs has been doubted because of assumed limited productivity compared to that in the Great Lakes. Despite this restraint, recent reports document adfluvial Chinook salmon residing in reservoirs ranging from 0.56 to 19.5 km^3^ in total water capacity (see Romer and Monzyk 2014; Perales et al. 2015 and citations therein). The reservoir created by Cougar Dam considered here is the smallest reservoir (0.27 km^3^), in which evidence for adfluvial Chinook salmon has emerged thus far. Perhaps it is not surprising the actual number of adfluvial Chinook salmon discovered is small. Additional information on the adfluvial life history tactic could improve our understanding of the role these fish may have in other reintroductions of Chinook salmon above dams.

We provide important information on the biology of the adfluvial Chinook salmon that collectively contributes toward improving our understanding of this rare life history tactic. We found evidence that unsampled males contributing to the population were significantly younger than females (Table 4). The Chinook salmon male precocial resident life history is well known (Quinn 2011), and it is therefore not surprising that several age 2 unsampled males had successfully reproduced. In addition, we did not identify any age 1 to age 3 adfluvial female Chinook salmon via grandparentage assignment, which is consistent with laboratory experiments showing that females cannot mature prior to age 3 (Taylor 1989). The lack of assignments identifying adfluvial females younger than age 4 also suggested a low false‐positive assignment rate. We did not observe any age 3 adfluvial Chinook salmon. However, few adults typically spawn at that age within the basin (Johnson and Friesen 2010). Unlike Romer and Monzyk (2014), we did not observe any ag 6 adults.

Complete age and reproductive success distributions for male and female adfluvial Chinook salmon in this system remain unknown for three reasons: (1) we did not have knowledge of all the mate pairs that occurred above the dam; (2) every single juvenile produced in the system was not sampled; and (3) the identification of any production by potential adfluvial–adfluvial grandparent pairs was not possible in this analysis. This lack of information may explain why 7 ± 4% of offspring in adult–juvenile pedigrees with one unassigned parent remained unexplained after testing all four hypotheses. Regardless, our genetic evidence that both adfluvial male and female Chinook salmon were successful at contributing to offspring improves our assessment of productivity for the reintroduced population above Cougar Dam.

We found that nonanadromous life history tactics slightly increase the estimates of CRR, assuming each unique grandparent pair that assigned to grandoffspring only produced one unsampled adult. The observed increases in CRR were not enough to meet replacement for either the 2007 or 2008 cohorts. Even with the incorporation of adfluvial Chinook salmon offspring, CRR for adults reintroduced above Cougar Dam are low compared to other published studies (Anderson et al. 2015; Evans et al. 2015). However, just as in parentage assignments, grandparentage methods are limited by genotyping error and further exacerbated by our conservative exclusion criterion, which could negatively bias our estimates. Our estimates for adfluvial Chinook salmon could also be negatively biased because grandparentage assignments do not account for unsampled adults that were not successful at reproducing and there may have been other grandparent pairs that we did not include in our analysis. Alternatively, estimates of adfluvial Chinook salmon may be positively biased because some unique Gtrios identified may be incorrect based on the expected number of false Gtrios calculated (Table 3). Results are more likely negatively biased because we would have expected multiple Gtrios assignments for unsampled mothers younger than age 3 if false‐positive assignment rates were high, which is not the case in this study. Despite these limitations with grandparentage assignment, our work is a first step towards understanding the degree adfluvial adults contribute to the demographic viability of Chinook salmon populations created by reintroduction programs.

Our approaches for testing hypotheses to explain unassigned parents are subject to some biases. SOLOMON only simulates uniform reproductive success distributions among parents, which does not realistically reflect Chinook salmon biology. The uniform reproductive success distribution could bias estimation of the percentage of offspring explained by genotyping error. This bias may be positive or negative depending on whether genotyping errors occurred in unsuccessful or highly fit adults, respectively, whereas genotyping error that occurred in an offspring is limited to that assignment. In addition, the current model for genotyping error in SOLOMON is random, rather than locus‐specific, which may also bias assignment rates. The direction of the bias likely depends on variation among locus‐specific genotyping error rates. Furthermore, our estimates of the percent of unassigned parents explained by incorrect sex identification of adults are also biased because some parent–offspring pairs, as well as putative incorrectly sexed adults, were excluded from the analyses due to the stringent requirements for the one‐parent known option in SOLOMON (Table 2). Our overall conclusions would not likely change with their inclusion because the excluded assignments represent a low proportion of the total number that could have been included in the analysis (Table 2). Collectively, our methods for testing the hypotheses may be negatively biased because 7 ± 4% of offspring in the adult–juvenile genetics pedigrees remain unexplained. However, our conclusions regarding the existence of the adfluvial life history is not affected by the bias because we often observed more Gtrios than expected by chance alone.

Grandparentage assignment methods can be broadly applied to any population that has been genetically pedigreed over multiple generations. In addition, grandparentage methods may be used with programs such as GERUD to infer the genotypes of the missing adults, assuming several variable loci are used and a sufficient number of offspring were sampled (Jones 2005). As genetic monitoring practices become more common, the application of grandparentage methodology will likely provide key insights into population productivity and connectivity, and perhaps identify other rare life history tactics.

## Conflict of Interest

None declared.

## Supporting information


**Table S1.** Summary of the number of juveniles collected in the screw trap, tissue sampled, and genotyped from 2009 to 2013.
**Table S2.** Summary of the number of juveniles used in the adult–juvenile genetic pedigrees from 2008 to 2013, genotyping error rate, the percent of offspring with one unassigned parent, the percent of offspring with one unassigned parent explained by four hypotheses (1) genotyping error, (2) missing anadromous adults, (3) incorrect sex identification of adults, and (4) missing adfluvial adults.
**Table S3.** Summary of the number of mate pairs identified in adult–adult and adult–juvenile genetic pedigrees assembled from 2007 to 2013.
**Table S4.** All grandoffspring assignments with unassigned mothers or fathers made to grandparent pairs for each adult–juvenile genetic pedigree from 2008 to 2013.

## References

[ece32378-bib-0001] Anderson, J. H. , G. R. Pess , R. W. Carmichael , M. J. Ford , T. D. Cooney , C. M. Baldwin , et al. 2014 Planning pacific salmon and steelhead reintroductions aimed at long‐term viability and recovery. N. Am. J. Fish. Manage 34:72–93.

[ece32378-bib-0002] Anderson, J. H. , P. Faulds , K. Burton , M. E. Koehler , W. I. Atlas , and T. J. Quinn . 2015 Dispersal and productivity of Chinook (*Oncorhynchus tshawytscha*) and coho (*Oncorhynchus kisutch*) salmon colonizing newly accessible habitat. Can. J. Fish Aquat. Sci. 72:454–465.

[ece32378-bib-0003] Banks, M. A. , M. S. Blouin , B. A. Baldwin , V. K. Rashbrook , H. A. Fitzgerald , S. M. Blankenship , et al. 1999 Isolation and inheritance of novel microsatellites in Chinook salmon (*Oncorhynchus tschawytscha*). J. Heredity 90:281–288.

[ece32378-bib-0004] Banks, M. A. , N. Sard , K. G. O'Malley , D. Jacobson , M. Hogansen , and M. A. Johnson 2014 A genetics‐based evaluation of the spring Chinook salmon reintroduction program above Cougar dam, South Fork McKenzie River, 2007–2013. U. S. Army Corps of Engineers, Portland District.

[ece32378-bib-0005] Benjamini, Y. , and Y. Hochberg . 1995 Controlling the false discovery rate: a practical and powerful approach to multiple testing. J. R. Stat. Soc. Ser. B Methodol. 57:289–300.

[ece32378-bib-0006] Boer, P. D. 1968 Spreading of risk and stabilization of animal numbers. Acta. Biotheor. 18:165–194. 4984481 10.1007/BF01556726

[ece32378-bib-0007] Botsford, L. W. , and J. G. Brittnacher . 1998 Viability of sacramento river winter‐run Chinook salmon. Conserv. Biol. 12:65–79.

[ece32378-bib-0008] Brunelli, J. P. , K. J. Wertzler , K. Sundin , and G. H. Thorgaard . 2008 Y‐specific sequences and polymorphisms in rainbow trout and Chinook salmon. Genome 51:739–748. 18772952 10.1139/G08-060

[ece32378-bib-0009] Cairney, M. , J. Taggart , and B. Høyheim . 2000 Characterization of microsatellite and minisatellite loci in Atlantic salmon (Salmo salar L.) and cross‐species amplification in other salmonids. Mol. Ecol. 9:2175–2178. 11123640

[ece32378-bib-0010] Chamberlin, T. C. 1965 The method of multiple working hypotheses. Science 148:754–759. 17748786 10.1126/science.148.3671.754

[ece32378-bib-0011] Christie, M. R. , M. L. Marine , and M. S. Blouin . 2011 Who are the missing parents? Grandparentage analysis identifies multiple sources of gene flow into a wild population. Mol. Ecol. 20:1263–1276. 21244538 10.1111/j.1365-294X.2010.04994.x

[ece32378-bib-0012] Christie, M. R. , J. A. Tennessen , and M. S. Blouin . 2013 Bayesian parentage analysis with systematic accountability of genotyping error, missing data and false matching. Bioinformatics 29:725–732. 23365409 10.1093/bioinformatics/btt039

[ece32378-bib-0013] Emery, L. 1985 Review of fish species introduced into the Great Lakes, 1819–1974. 1451 Green Road Ann Arbor, Michigan 48 105: Great Lakes Fishery Commission, Ann Arbor, Michigan, USA.

[ece32378-bib-0014] Evans, M. L. , M. A. Johnson , D. Jacobson , J. Wang , M. Hogansen , and K. G. O'Malley . 2015 Evaluating a multi‐generational retinroduction program for threatened salmon using genetic parentage analysis. Can. J. Fish Aquat. Sci. 73:844–852.

[ece32378-bib-0015] Figge, F. 2004 Bio‐folio: applying portfolio theory to biodiversity. Biodivers. Conserv. 13:827–849.

[ece32378-bib-0016] Ford, M. , T. N. Pearsons , and A. Murdoch . 2015 The spawning success of early maturing resident hatchery Chinook salmon in a natural river system. Trans. Am. Fish. Soc. 144:539–548.

[ece32378-bib-0017] Fox, G. A. 2005 Extinction risk of heterogeneous populations. Ecology 86:1191–1198.

[ece32378-bib-0018] Greene, C. M. , J. E. Hall , K. R. Guilbault , and T. P. Quinn . 2009 Improved viability of populations with diverse life‐history portfolios. Biol. Lett. 6:rsbl20090780. 10.1098/rsbl.2009.0780 PMC2880035 20007162

[ece32378-bib-0019] Greig, C. , D. P. Jacobson , and M. A. Banks . 2003 New tetranucleotide microsatellites for fine‐scale discrimination among endangered Chinook salmon (*Oncorhynchus tshawytscha*). Mol. Ecol. Notes 3:376–379.

[ece32378-bib-0020] Ivanova, N. V. , J. R. Dewaard , and P. D. N. Hebert . 2006 An inexpensive, automation‐friendly protocol for recovering high‐quality DNA. Mol. Ecol. Notes 6:998–1002.

[ece32378-bib-0021] Johnson, M. A. , and T. G. Friesen . 2010 Spring Chinook salmon Hatcheries in the Willamette Basin: Existing Data, Discernable Patterns and Information Gaps. Pp1–87. U.S. Army Corps of Engineers, Portland District: Oregon State Department of Fish and Wildlife.

[ece32378-bib-0022] Jones, A. G. 2005 gerud 2.0: a computer program for the reconstruction of parental genotypes from half‐sib progeny arrays with known or unknown parents. Mol. Ecol. Notes 5:708–711.

[ece32378-bib-0023] Jones, O. R. , and J. Wang . 2010 COLONY: a program for parentage and sibship inference from multilocus genotype data. Mol. Ecol. Res. 10:551–555. 10.1111/j.1755-0998.2009.02787.x 21565056

[ece32378-bib-0024] Kalinowski, S. T. , M. L. Taper , and T. C. Marshall . 2007 Revising how the computer program CERVUS accommodates genotyping error increases success in paternity assignment. Mol. Ecol. 16:1099–1106. 17305863 10.1111/j.1365-294X.2007.03089.x

[ece32378-bib-0025] Koellner, T. , and O. J. Schmitz . 2006 Biodiversity, ecosystem function, and investment risk. Bioscience 56:977–985.

[ece32378-bib-0026] Letcher, B. H. , and T. L. King . 2001 Parentage and grandparentage assignment with known and unknown matings: application to Connecticut River Atlantic salmon restoration. Can. J. Fish Aquat. Sci. 58:1812–1821.

[ece32378-bib-0027] Marshall, T. C. , J. Slate , L. E. Kruuk , and J. M. Pemberton . 1998 Statistical confidence for likelihood‐based paternity inference in natural populations. Mol. Ecol. 7:639–655. 9633105 10.1046/j.1365-294x.1998.00374.x

[ece32378-bib-0028] Naish, K. A. , and L. Park . 2002 Linkage relationships for 35 new microsatellite loci in Chinook salmon *Oncorhynchus tshawytscha* . Anim. Genet. 33:312–327. 10.1046/j.1365-2052.2002.t01-4-00886.x 12139517

[ece32378-bib-0029] NMFS . 2005 Endangered and threatened species: final listing determinations for 16 ESUs of West Coast salmon, and final 4(d) protective regulations for threatened salmonid ESUs.). Federal Register 70:123(28 June 2005):37160–37204.: National Marine Fisheries Service.

[ece32378-bib-0030] NMFS . 2008 Endangered Species Act Section 7 (a)(2) Consultation Supplemental Biological Opinion & Magnuson‐Stevens Fishery Conservation & Management Act Essential Fish Habitat Consultation.). NOAA's National Marine Fisheries Service (NMFS) Northwest Region.

[ece32378-bib-0031] ODFW, NMFS . 2010 Upper Willamette conservation and recovery plan for Chinook salmon and Steelhead.). ODFW, Salem.

[ece32378-bib-0032] Olsen, J. B. , P. Bentzen , and J. Seeb . 1998 Characterization of seven microsatellite loci derived from pink salmon. Mol. Ecol. 7:1087–1089. 9711869

[ece32378-bib-0033] Perales, K. M. , J. Rowan , and P. B. Moyle . 2015 Evidence of landlocked Chinook salmon populations in California. N. Am. J. Fish. Manage 35:1101–1105.

[ece32378-bib-0034] Quinn, T. P. 2011 The behavior and ecology of Pacific salmon and trout. UBC Press, University of Washington, Seattle, WA.

[ece32378-bib-0035] R Core Team . 2014 R: a language and environment for statistical computing. R Foundation for Statistical Computing, Vienna, Austria.

[ece32378-bib-0036] Ricker, W. 1938 “Residual” and kokanee salmon in Cultus lake. J. Fish. Board Canada 4:192–218.

[ece32378-bib-0037] Robert, A. , B. Colas , I. Guigon , C. Kerbiriou , J. B. Mihoub , M. Saint‐Jalme , et al. 2015 Defining reintroduction success using IUCN criteria for threatened species: a demographic assessment. Anim. Conserv. Early View 18:397–406.

[ece32378-bib-0038] Rohde, J. , K. L. Fresh , and T. P. Quinn . 2014 Factors affecting partial migration in Puget sound Coho salmon. N. Am. J. Fish. Manage 34:559–570.

[ece32378-bib-0039] Romer, J. D. , and F. R. Monzyk . 2014 Adfluvial life history in spring Chinook salmon from Quartzville creek, Oregon. N. Am. J. Fish. Manage 34:885–891.

[ece32378-bib-0040] Rubio de Casas, R. , K. Donohue , D. L. Venable , and P. O. Cheptou . 2015 Gene‐flow through space and time. Evol. Ecol. 29:813–831.

[ece32378-bib-0041] Sard, N. M. , K. G. O'Malley , D. P. Jacobson , M. J. Hogansen , M. A. Johnson , and M. A. Banks . 2015 Factors influencing spawner success in a spring Chinook salmon (*Oncorhynchus tshawytscha*) reintroduction program. Can. J. Fish Aquat. Sci. 72:1390–1397.

[ece32378-bib-0042] Sard, N. M. , M. A. Johnson , D. P. Jacobson , M. Hogansen , K. G. O'Malley , and M. A. Banks . 2016 Genetic monitoring guides adaptive management of a migratory fish reintroduction. Anim. Conserv. Early View form: http://onlinelibrary.wiley.com/doi/10.1111/acv.12278/full.

[ece32378-bib-0043] Taylor, E. B. 1989 Precocial male maturation in laboratory‐reared populations of Chinook salmon, *Oncorhynchus tshawytscha* . Can. J. Zool. 67:1665–1669.

[ece32378-bib-0044] Weaver, J. L. , P. C. Paquet , and L. F. Ruggiero . 1996 Resilience and conservation of large carnivores in the Rocky Mountains. Conserv. Biol. 10:964–976.

[ece32378-bib-0045] Williamson, K. S. , J. F. Cordes , and B. May . 2002 Characterization of microsatellite loci in Chinook salmon (*Oncorhynchus tshawytscha*) and cross‐species amplification in other salmonids. Mol. Ecol. 2:17–19.

[ece32378-bib-0046] Zymonas, N. D. , J. V. Tranquilli , and M. Hogansen . 2010 Monitoring and Evaluation of Impacts to Bull Trout (Salvelinus confluentus) and Spring Chinook (Oncorhynchus tshawytscha) in the South Fork McKenzie River from Construction of Water Temperature Control Facilities at Cougar Dam, Oregon. *U.S Army Corps of Engineers, Project Number: W66QKZ13186766, Oregon Department of Fisheries and Wildlife*.

