# Supplementary material for: Grandparentage assignments identify unexpected adfluvial life history tactic contributing offspring to a reintroduced population
Source: Ecol Evol. 2016 Aug 26;6(19):6773–83. doi: 10.1002/ece3.2378 (PMC5513239; doi:10.1002/ece3.2378)
Supplement: Supplementary file 1 — Table S1. Summary of the number of juveniles collected in the screw trap, tissue sampled, and genotyped from 2009 to 2013. Table S2. Summary of the number of juveniles used in the adult–juvenile genetic pedigrees from 2008 to 2013, genotyping error rate, the percent of offspring with one unassigned parent, the percent of offspring with one unassigned parent explained by four hypotheses (1) genotyping error, (2) missing anadromous adults, (3) incorrect sex identification of adults, and (4) missing adfluvial adults. Table S3. Summary of the number of mate pairs identified in adult–adult and adult–juvenile genetic pedigrees assembled from 2007 to 2013. Table S4. All grandoffspring assignments with unassigned mothers or fathers made to grandparent pairs for each adult–juvenile genetic pedigree from 2008 to 2013. [file ECE3-6-6773-s001.docx]

Table S1: Summary of the number of juveniles collected in the screw trap, tissue sampled, and genotyped from 2009-2013.

| **Year** | **Screw trap** | **Tissue sampled** | **Genotyped** |
| --- | --- | --- | --- |
| 2009 | 11024 | 5916 | 2000 |
| 2010 | 28150 | 7961 | 1826 |
| 2011 | 4439 | 4141 | 2035 |
| 2012 | 6754 | 4995 | 2220 |
| 2013 | 20212 | 8485 | 2792 |
| 2014 | 11438 | 5878 | 2087 |

Table S2: Summary of the number of juveniles used in the adult-juvenile genetic pedigrees from 2008-2013, genotyping error rate, the percent of offspring with one unassigned parent, the percent of offspring with one unassigned parent explained by four hypotheses (1) genotyping error, (2) missing anadromous adults, (3) incorrect sex identification of adults, and (4) missing adfluvial adults.

| **Parent year** | **Genotyping error (%)** | **Percent offspring with one unassigned parent** | **Hypotheses (% explained)** | | | | |
| --- | --- | --- | --- | --- | --- | --- | --- |
|  |  |  | **1** | **2** | **3** | **4** | **Total** |
| 2008 | 2 | 12.5 | 7.2 | 0.0 | 0.1 | 0.0 | 7.3 |
| 2009 | 4 | 24.7 | 19 | 0.4 | 0.3 | 0.2 | 19.3 |
| 2010 | 2 | 20.6 | 6.8 | 2.9 | 0.9 | 0.9 | 11.5 |
| 2011 | 2 | 26.4 | 6.6 | 1.9 | 0.2 | 2.4 | 11.1 |
| 2012 | 1 | 15.2 | 2.8 | 0.5 | 0.2 | 5.5 | 9.0 |
| 2013 | 1 | 8.0 | 2.1 | 0.5 | 0.2 | 2.6 | 5.5 |

Table S3. Summary of the number of mate pairs identified in adult-adult and adult-juvenile genetic pedigrees assembled from 2007-2013.

| **Year** | **Adult-Juvenile** | **Adult-Adult** |
| --- | --- | --- |
| 2007 | 0 | 173 |
| 2008 | 476 | 33 |
| 2009 | 576 | 11 |
| 2010 | 258 | 1 |
| 2011 | 300 | 0 |
| 2012 | 615 | 0 |
| 2013 | 391 | 0 |
|  |  |  |

Table S4. All grandoffspring assignments with unassigned mothers or fathers made to grandparent pairs for each adult-juvenile genetic pedigree from 2008-2013. The inferred age of the unsampled parent is included. Whether assignments still remained (Yes/No) after genotyping the grandparent pair grandoffspring trios at an additional 4 loci is also indicated (15 loci in total).

| **Pedigree** | **Unassigned parent** | **Age** | **Grandmother** | **Grandfather** | **Grandoffspring** | **15 Loci** |
| --- | --- | --- | --- | --- | --- | --- |
| 2008 | Father | 1 | MR07_041 | MR07_237 | MR09J_5_18_036 | Yes |
| 2010 | Father | 1 | MR09A_0157 | MR09A_0002 | MR11J_04_28_027 | Yes |
| 2010 | Father | 1 | MR09A_0157 | MR09A_0002 | MR11J_05_02_002 | Yes |
| 2009 | Father | 2 | MR07_127 | MR07_378 | MR10J_04_24_013^a^ | No |
| 2009 | Father | 2 | MR07_840 | MR07_845 | MR10J_04_23_023 | Yes |
| 2009 | Father | 2 | MR07_814 | MR07_879 | MR10J_05_19_018 | Yes |
| 2010 | Father | 2 | MR08_343 | MR08_602 | MR11J_03_12_001 | Yes |
| 2010 | Father | 2 | MR08_364 | MR08_559 | MR11J_03_21_009 | Yes |
| 2010 | Father | 2 | MR08_196 | MR08_689 | MR11J_04_11_026 | Yes |
| 2010 | Father | 2 | MR08_102 | MR08_607 | MR11J_04_25_036 | Yes |
| 2010 | Father | 2 | MR08_102 | MR08_607 | MR11J_04_25_039 | Yes |
| 2010 | Father | 2 | MR08_343 | MR08_602 | MR11J_04_25_065 | Yes |
| 2010 | Father | 2 | MR08_277 | MR08_601 | MR11J_05_02_053 | Yes |
| 2010 | Father | 2 | MR08_343 | MR08_602 | MR11J_05_05_027 | Yes |
| 2010 | Father | 2 | MR08_699 | MR08_612 | MR11J_05_12_017 | Yes |
| 2010 | Father | 2 | MR08_680 | MR08_125 | MR11J_05_17_003 | Yes |
| 2010 | Father | 2 | MR08_699 | MR08_612 | MR11J_05_23_019 | Yes |
| 2010 | Father | 2 | MR08_812 | MR08_698 | MR11J_05_23_023 | Yes |
| 2010 | Father | 2 | MR08_812 | MR08_698 | MR11J_05_23_096 | Yes |
| 2010 | Father | 2 | MR08_699 | MR08_612 | MR11J_05_31_028 | Yes |
| 2010 | Father | 2 | MR08_102 | MR08_607 | MR11J_08_12_005 | Yes |
| 2010 | Father | 2 | MR08_329 | MR08_125 | MR11J_09_23_009 | Yes |
| 2010 | Father | 2 | MR08_733 | MR08_559 | MR11J_10_05_002 | Yes |
| 2011 | Father | 2 | MR09A_0470 | MR09A_0095 | MR12J_05_07_028^ab^ | No |
| 2011 | Father | 2 | MR09A_0470 | MR09A_0065 | MR12J_05_07_028^b^ | Yes |
| 2011 | Father | 2 | MR09A_0470 | MR09A_0065 | MR12J_05_15_012^b^ | Yes |
| 2011 | Father | 2 | MR09A_0470 | MR09A_0095 | MR12J_05_15_012^b^ | Yes |
| 2011 | Father | 2 | MR09A_0510 | MR09A_0775 | MR12J_05_16_006^c^ | Yes |
| 2011 | Father | 2 | MR09A_0510 | MR09A_0775 | MR12J_05_16_054^c^ | Yes |
| 2011 | Father | 2 | MR09A_0510 | MR09A_0775 | MR12J_05_17_009^c^ | Yes |
| 2011 | Father | 2 | MR09A_0470 | MR09A_0095 | MR12J_05_14_111^a^ | No |
| 2011 | Father | 2 | MR09A_0470 | MR09A_0095 | MR12J_05_15_040^a^ | No |
| 2011 | Father | 2 | MR09A_0470 | MR09A_0095 | MR12J_05_21_075^a^ | No |
| 2011 | Father | 2 | MR09A_0664 | MR09A_0615 | MR12J_04_13_024 | Yes |
| 2011 | Father | 2 | MR09A_0664 | MR09A_0615 | MR12J_04_13_026 | Yes |
| 2011 | Father | 2 | MR09A_0664 | MR09A_0615 | MR12J_04_13_033 | Yes |
| 2011 | Father | 2 | MR09A_0664 | MR09A_0615 | MR12J_04_16_075 | Yes |
| 2011 | Father | 2 | MR09A_0470 | MR09A_0095 | MR12J_05_17_014 | Yes |
| 2011 | Father | 2 | MR09A_0624 | MR09A_0215 | MR12J_09_07_001 | Yes |
| 2012 | Father | 2 | MR10A_045 | MR10A_156 | MR13J_04_22_1351^bc^ | No |
| 2012 | Father | 2 | MR10A_273 | MR10TH_149 | MR13J_04_02_0408 | Yes |
| 2012 | Father | 2 | MR10A_273 | MR10TH_149 | MR13J_04_14_0873 | Yes |
| 2012 | Father | 2 | MR10A_250 | MR10TH_109 | MR13J_04_15_0967 | Yes |
| 2012 | Father | 2 | MR10A_250 | MR10TH_109 | MR13J_04_20_1214 | Yes |
| 2012 | Father | 2 | MR10A_451 | MR10A_455 | MR13J_04_27_1556 | Yes |
| 2012 | Father | 2 | MR10A_451 | MR10A_455 | MR13J_04_29_1718 | Yes |
| 2012 | Father | 2 | MR10TH_244 | MR10TH_038 | MR13J_05_28_2677 | Yes |
| 2012 | Father | 2 | MR10A_273 | MR10TH_149 | MR13J_05_31_2743 | Yes |
| 2013 | Father | 2 | MR11_002 | MR11_204 | MR14J_06_02_006^b^ | Yes |
| 2013 | Father | 2 | MR11_040 | MR11_204 | MR14J_06_02_006^b^ | Yes |
| 2013 | Father | 2 | MR11_040 | MR11_085 | MR14J_05_03_015^a^ | No |
| 2013 | Father | 2 | MR11_031 | MR11_069 | MR14J_05_18_092^a^ | No |
| 2013 | Father | 2 | MR11_226 | MR11_005 | MR14J_04_11_026^a^ | No |
| 2013 | Father | 2 | MR11_226 | MR11_005 | MR14J_07_11_002^a^ | No |
| 2013 | Father | 2 | MR11_002 | MR11_204 | MR14J_05_29_006^a^ | No |
| 2013 | Father | 2 | MR11_002 | MR11_204 | MR14J_05_31_006^a^ | No |
| 2013 | Father | 2 | MR11_186 | MR11_351 | MR14J_04_04_003 | Yes |
| 2013 | Father | 2 | MR11A_345 | MR11A_228 | MR14J_04_09_017 | Yes |
| 2013 | Father | 2 | MR11_186 | MR11_351 | MR14J_04_11_011 | Yes |
| 2013 | Father | 2 | MR11A_345 | MR11A_301 | MR14J_04_30_018 | Yes |
| 2013 | Father | 2 | MR11A_345 | MR11A_228 | MR14J_05_02_005 | No |
| 2013 | Father | 2 | MR11_040 | MR11_204 | MR14J_05_05_047 | Yes |
| 2013 | Father | 2 | MR11_226 | MR11_005 | MR14J_05_08_015 | Yes |
| 2013 | Father | 2 | MR11_186 | MR11_351 | MR14J_05_08_051 | Yes |
| 2013 | Father | 2 | MR11A_345 | MR11A_031 | MR14J_05_14_054 | Yes |
| 2013 | Father | 2 | MR11A_345 | MR11A_031 | MR14J_05_14_097 | Yes |
| 2013 | Father | 2 | MR11_002 | MR11_204 | MR14J_05_16_051 | Yes |
| 2013 | Father | 2 | MR11_025 | MR11A_267 | MR14J_05_19_041 | Yes |
| 2013 | Father | 2 | MR11A_345 | MR11A_301 | MR14J_05_29_033 | Yes |
| 2013 | Father | 2 | MR11_333 | MR11_167 | MR14J_05_29_045 | Yes |
| 2013 | Father | 2 | MR11_002 | MR11_204 | MR14J_05_31_010 | Yes |
| 2013 | Father | 2 | MR11A_345 | MR11A_031 | MR14J_06_25_001 | Yes |
| 2011 | Father | 4 | MR07_125 | MR07_260 | MR12J_04_11_048 | Yes |
| 2011 | Father | 4 | MR07_147 | MR07_575 | MR12J_04_23_033 | Yes |
| 2011 | Father | 4 | MR07_147 | MR07_575 | MR12J_04_23_039 | Yes |
| 2011 | Father | 4 | MR07_389 | MR07_408 | MR12J_05_04_007 | Yes |
| 2011 | Father | 4 | MR07_454 | MR07_649 | MR12J_05_15_034 | Yes |
| 2011 | Father | 4 | MR07_147 | MR07_595 | MR12J_05_16_023 | Yes |
| 2011 | Father | 4 | MR07_832 | MR07_561 | MR12J_05_21_064 | Yes |
| 2011 | Father | 4 | MR07_456 | MR07_279 | MR12J_05_23_011 | Yes |
| 2011 | Father | 4 | MR07_147 | MR07_575 | MR12J_07_02_004 | Yes |
| 2012 | Father | 4 | MR08_042 | MR08_585 | MR13J_04_17_0998^b^ | Yes |
| 2012 | Father | 4 | MR08_042 | MR08_664 | MR13J_04_17_0998^b^ | Yes |
| 2012 | Father | 4 | MR08_042 | MR08_585 | MR13J_04_18_1116^b^ | Yes |
| 2012 | Father | 4 | MR08_042 | MR08_664 | MR13J_04_18_1116^b^ | Yes |
| 2012 | Father | 4 | MR08_042 | MR08_585 | MR13J_05_01_1784^b^ | Yes |
| 2012 | Father | 4 | MR08_042 | MR08_664 | MR13J_05_01_1784^b^ | Yes |
| 2012 | Father | 4 | MR08_042 | MR08_585 | MR13J_05_01_1795^b^ | No |
| 2012 | Father | 4 | MR08_042 | MR08_664 | MR13J_05_01_1795^b^ | Yes |
| 2012 | Father | 4 | MR08_042 | MR08_585 | MR13J_05_01_1800^b^ | Yes |
| 2012 | Father | 4 | MR08_042 | MR08_664 | MR13J_05_01_1800^b^ | Yes |
| 2012 | Father | 4 | MR08_042 | MR08_585 | MR13J_05_03_1902^b^ | Yes |
| 2012 | Father | 4 | MR08_042 | MR08_664 | MR13J_05_03_1902^b^ | Yes |
| 2012 | Father | 4 | MR08_042 | MR08_585 | MR13J_05_08_2084^b^ | Yes |
| 2012 | Father | 4 | MR08_042 | MR08_664 | MR13J_05_08_2084^b^ | Yes |
| 2012 | Father | 4 | MR08_042 | MR08_585 | MR13J_05_10_2164^b^ | Yes |
| 2012 | Father | 4 | MR08_042 | MR08_664 | MR13J_05_10_2164^b^ | Yes |
| 2012 | Father | 4 | MR08_721 | MR08_620 | MR13J_04_04_0498 | Yes |
| 2012 | Father | 4 | MR08_042 | MR08_664 | MR13J_04_06_0608 | Yes |
| 2012 | Father | 4 | MR08_042 | MR08_664 | MR13J_04_14_0901 | Yes |
| 2012 | Father | 4 | MR08_042 | MR08_664 | MR13J_04_17_1018 | Yes |
| 2012 | Father | 4 | MR08_042 | MR08_664 | MR13J_04_20_1177 | Yes |
| 2012 | Father | 4 | MR08_739 | MR08_606 | MR13J_04_20_1192 | Yes |
| 2012 | Father | 4 | MR08_042 | MR08_664 | MR13J_04_20_1222 | Yes |
| 2012 | Father | 4 | MR08_042 | MR08_664 | MR13J_04_22_1260 | Yes |
| 2012 | Father | 4 | MR08_042 | MR08_664 | MR13J_04_22_1277 | Yes |
| 2012 | Father | 4 | MR08_042 | MR08_664 | MR13J_04_27_1588 | Yes |
| 2012 | Father | 4 | MR08_144 | MR08_256 | MR13J_04_29_1710 | Yes |
| 2012 | Father | 4 | MR08_042 | MR08_664 | MR13J_05_01_1789 | Yes |
| 2012 | Father | 4 | MR08_042 | MR08_664 | MR13J_05_06_1926 | Yes |
| 2012 | Father | 4 | MR08_042 | MR08_664 | MR13J_05_06_1940 | Yes |
| 2012 | Father | 4 | MR08_042 | MR08_664 | MR13J_05_06_1952 | Yes |
| 2012 | Father | 4 | MR08_042 | MR08_664 | MR13J_05_06_2020 | Yes |
| 2012 | Father | 4 | MR08_042 | MR08_664 | MR13J_05_10_2112 | Yes |
| 2012 | Father | 4 | MR08_042 | MR08_664 | MR13J_05_13_2282 | Yes |
| 2012 | Father | 4 | MR08_042 | MR08_664 | MR13J_05_31_2727 | Yes |
| 2012 | Father | 4 | MR08_042 | MR08_664 | MR13J_08_12_2834 | Yes |
| 2012 | Father | 4 | MR08_042 | MR08_664 | MR13J_08_14_2839 | Yes |
| 2012 | Father | 4 | MR08_042 | MR08_664 | MR13J_08_14_2840 | Yes |
| 2013 | Father | 4 | MR09A_1193 | MR09A_0595 | MR14J_03_02_002 | Yes |
| 2011 | Mother | 4 | MR07_024 | MR07_404 | MR12J_04_23_051^b^ | Yes |
| 2011 | Mother | 4 | MR07_261 | MR07_404 | MR12J_04_23_051^b^ | Yes |
| 2011 | Mother | 4 | MR07_071 | MR07_149 | MR12J_04_13_010^a^ | No |
| 2011 | Mother | 4 | MR07_071 | MR07_149 | MR12J_04_13_020^a^ | No |
| 2011 | Mother | 4 | MR07_155 | MR07_434 | MR12J_04_11_019 | Yes |
| 2011 | Mother | 4 | MR07_155 | MR07_434 | MR12J_04_11_036 | Yes |
| 2011 | Mother | 4 | MR07_155 | MR07_434 | MR12J_04_12_012 | Yes |
| 2011 | Mother | 4 | MR07_071 | MR07_149 | MR12J_04_13_037 | Yes |
| 2011 | Mother | 4 | MR07_155 | MR07_434 | MR12J_04_16_007 | Yes |
| 2011 | Mother | 4 | MR07_155 | MR07_434 | MR12J_04_16_011 | Yes |
| 2011 | Mother | 4 | MR07_155 | MR07_434 | MR12J_04_16_023 | Yes |
| 2011 | Mother | 4 | MR07_155 | MR07_434 | MR12J_04_16_050 | Yes |
| 2011 | Mother | 4 | MR07_071 | MR07_149 | MR12J_04_16_065 | Yes |
| 2011 | Mother | 4 | MR07_488 | MR07_074 | MR12J_04_16_081 | Yes |
| 2011 | Mother | 4 | MR07_071 | MR07_149 | MR12J_04_16_094 | Yes |
| 2011 | Mother | 4 | MR07_071 | MR07_149 | MR12J_04_17_014 | Yes |
| 2011 | Mother | 4 | MR07_024 | MR07_404 | MR12J_04_18_008 | Yes |
| 2011 | Mother | 4 | MR07_155 | MR07_434 | MR12J_04_19_008 | Yes |
| 2011 | Mother | 4 | MR07_024 | MR07_404 | MR12J_04_23_002 | Yes |
| 2011 | Mother | 4 | MR07_147 | MR07_010 | MR12J_04_23_022 | Yes |
| 2011 | Mother | 4 | MR07_439 | MR07_542 | MR12J_04_23_042 | Yes |
| 2011 | Mother | 4 | MR07_024 | MR07_404 | MR12J_04_23_071 | Yes |
| 2011 | Mother | 4 | MR07_024 | MR07_404 | MR12J_05_04_004 | Yes |
| 2011 | Mother | 4 | MR07_098 | MR07_660 | MR12J_05_17_017 | Yes |
| 2011 | Mother | 4 | MR07_098 | MR07_660 | MR12J_05_17_022 | Yes |
| 2011 | Mother | 4 | MR07_098 | MR07_660 | MR12J_05_18_029 | Yes |
| 2011 | Mother | 4 | MR07_213 | MR07_544 | MR12J_05_18_057 | Yes |
| 2011 | Mother | 4 | MR07_213 | MR07_544 | MR12J_05_22_027 | Yes |
| 2011 | Mother | 4 | MR07_213 | MR07_544 | MR12J_05_22_050 | Yes |
| 2012 | Mother | 4 | MR08_028 | MR08_677 | MR13J_04_22_1281^b^ | No |
| 2012 | Mother | 4 | MR08_028 | MR08_522 | MR13J_04_22_1281^b^ | Yes |
| 2012 | Mother | 4 | MR08_028 | MR08_677 | MR13J_04_22_1328^b^ | Yes |
| 2012 | Mother | 4 | MR08_028 | MR08_660 | MR13J_04_22_1328^b^ | Yes |
| 2012 | Mother | 4 | MR08_028 | MR08_522 | MR13J_04_22_1328^b^ | Yes |
| 2012 | Mother | 4 | MR08_028 | MR08_677 | MR13J_04_25_1504^b^ | Yes |
| 2012 | Mother | 4 | MR08_028 | MR08_660 | MR13J_04_25_1504^b^ | Yes |
| 2012 | Mother | 4 | MR08_028 | MR08_522 | MR13J_04_25_1504^b^ | Yes |
| 2012 | Mother | 4 | MR08_688 | MR08_631 | MR13J_04_27_1633^a^ | No |
| 2012 | Mother | 4 | MR08_394 | MR08_501 | MR13J_03_25_0138 | Yes |
| 2012 | Mother | 4 | MR08_688 | MR08_631 | MR13J_03_30_0222 | Yes |
| 2012 | Mother | 4 | MR08_688 | MR08_631 | MR13J_03_30_0235 | Yes |
| 2012 | Mother | 4 | MR08_688 | MR08_631 | MR13J_04_01_0251 | Yes |
| 2012 | Mother | 4 | MR08_688 | MR08_631 | MR13J_04_01_0263 | Yes |
| 2012 | Mother | 4 | MR08_688 | MR08_631 | MR13J_04_01_0318 | Yes |
| 2012 | Mother | 4 | MR08_688 | MR08_631 | MR13J_04_03_0410 | Yes |
| 2012 | Mother | 4 | MR08_688 | MR08_631 | MR13J_04_03_0451 | Yes |
| 2012 | Mother | 4 | MR08_726 | MR08_361 | MR13J_04_03_0457 | Yes |
| 2012 | Mother | 4 | MR08_688 | MR08_631 | MR13J_04_03_0463 | Yes |
| 2012 | Mother | 4 | MR08_699 | MR08_313 | MR13J_04_05_0561 | Yes |
| 2012 | Mother | 4 | MR08_697 | MR08_314 | MR13J_04_11_0679 | Yes |
| 2012 | Mother | 4 | MR08_726 | MR08_361 | MR13J_04_12_0807 | Yes |
| 2012 | Mother | 4 | MR08_028 | MR08_243 | MR13J_04_12_0808 | Yes |
| 2012 | Mother | 4 | MR08_688 | MR08_631 | MR13J_04_14_0940 | Yes |
| 2012 | Mother | 4 | MR08_739 | MR08_606 | MR13J_04_17_1061 | Yes |
| 2012 | Mother | 4 | MR08_697 | MR08_314 | MR13J_04_17_1079 | Yes |
| 2012 | Mother | 4 | MR08_739 | MR08_606 | MR13J_04_17_1085 | Yes |
| 2012 | Mother | 4 | MR08_739 | MR08_606 | MR13J_04_18_1099 | Yes |
| 2012 | Mother | 4 | MR08_726 | MR08_361 | MR13J_04_18_1117 | Yes |
| 2012 | Mother | 4 | MR08_726 | MR08_361 | MR13J_04_20_1161 | Yes |
| 2012 | Mother | 4 | MR08_726 | MR08_361 | MR13J_04_20_1255 | Yes |
| 2012 | Mother | 4 | MR08_688 | MR08_631 | MR13J_04_22_1282 | Yes |
| 2012 | Mother | 4 | MR08_080 | MR08_017 | MR13J_04_22_1285 | Yes |
| 2012 | Mother | 4 | MR08_688 | MR08_631 | MR13J_04_22_1341 | Yes |
| 2012 | Mother | 4 | MR08_028 | MR08_522 | MR13J_04_25_1481 | Yes |
| 2012 | Mother | 4 | MR08_028 | MR08_522 | MR13J_04_25_1497 | Yes |
| 2012 | Mother | 4 | MR08_028 | MR08_522 | MR13J_04_25_1503 | Yes |
| 2012 | Mother | 4 | MR08_028 | MR08_522 | MR13J_04_25_1513 | Yes |
| 2012 | Mother | 4 | MR08_028 | MR08_522 | MR13J_04_25_1531 | Yes |
| 2012 | Mother | 4 | MR08_378 | MR08_458 | MR13J_04_29_1750 | Yes |
| 2012 | Mother | 4 | MR08_144 | MR08_256 | MR13J_05_06_1963 | Yes |
| 2012 | Mother | 4 | MR08_144 | MR08_256 | MR13J_05_08_2032 | Yes |
| 2012 | Mother | 4 | MR08_144 | MR08_256 | MR13J_05_08_2050 | Yes |
| 2012 | Mother | 4 | MR08_688 | MR08_631 | MR13J_05_28_2672 | Yes |
| 2012 | Mother | 4 | MR08_366 | MR08_662 | MR13J_05_31_2733 | Yes |
| 2013 | Mother | 4 | MR09A_0738 | MR09A_0428 | MR14J_04_23_006^a^ | No |
| 2013 | Mother | 4 | MR09A_0738 | MR09A_0428 | MR14J_04_21_012 | Yes |
| 2013 | Mother | 4 | MR09A_0738 | MR09A_0428 | MR14J_04_21_028 | Yes |
| 2013 | Mother | 4 | MR09A_0738 | MR09A_0428 | MR14J_04_22_004 | Yes |
| 2013 | Mother | 4 | MR09A_0738 | MR09A_0428 | MR14J_04_22_005 | Yes |
| 2013 | Mother | 4 | MR09A_0738 | MR09A_0428 | MR14J_04_23_008 | Yes |
| 2013 | Mother | 4 | MR09A_0738 | MR09A_0428 | MR14J_04_23_012 | Yes |
| 2013 | Mother | 4 | MR09A_0738 | MR09A_0428 | MR14J_04_23_016 | Yes |
| 2013 | Mother | 4 | MR09A_0738 | MR09A_0428 | MR14J_04_24_011 | Yes |
| 2013 | Mother | 4 | MR09A_0738 | MR09A_0428 | MR14J_04_24_015 | Yes |
| 2013 | Mother | 4 | MR09A_0738 | MR09A_0428 | MR14J_04_24_016 | Yes |
| 2013 | Mother | 4 | MR09A_0738 | MR09A_0428 | MR14J_04_24_028 | Yes |
| 2013 | Mother | 4 | MR09A_0738 | MR09A_0428 | MR14J_04_26_003 | Yes |
| 2013 | Mother | 4 | MR09A_0738 | MR09A_0428 | MR14J_04_26_006 | Yes |
| 2013 | Mother | 4 | MR09A_0738 | MR09A_0428 | MR14J_04_26_012 | Yes |
| 2013 | Mother | 4 | MR09A_0738 | MR09A_0428 | MR14J_04_26_016 | Yes |
| 2013 | Mother | 4 | MR09A_0738 | MR09A_0428 | MR14J_04_27_007 | Yes |
| 2013 | Mother | 4 | MR09A_0738 | MR09A_0428 | MR14J_04_27_014 | Yes |
| 2013 | Mother | 4 | MR09A_0738 | MR09A_0428 | MR14J_04_28_004 | Yes |
| 2013 | Mother | 4 | MR09A_0738 | MR09A_0428 | MR14J_04_28_008 | Yes |
| 2013 | Mother | 4 | MR09A_0738 | MR09A_0428 | MR14J_04_28_009 | Yes |
| 2013 | Mother | 4 | MR09A_0738 | MR09A_0428 | MR14J_04_28_020 | Yes |
| 2013 | Mother | 4 | MR09A_0738 | MR09A_0428 | MR14J_04_28_024 | Yes |
| 2013 | Mother | 4 | MR09A_0738 | MR09A_0428 | MR14J_04_28_026 | Yes |
| 2013 | Mother | 4 | MR09A_0738 | MR09A_0428 | MR14J_04_28_030 | Yes |
| 2013 | Mother | 4 | MR09A_0738 | MR09A_0428 | MR14J_05_02_020 | Yes |
| 2013 | Mother | 4 | MR09A_0738 | MR09A_0428 | MR14J_05_02_045 | Yes |
| 2013 | Mother | 4 | MR09A_0738 | MR09A_0428 | MR14J_05_02_050 | Yes |
| 2012 | Father | 5 | MR07_103 | MR07_122 | MR13J_04_03_0431^b^ | No |
| 2012 | Father | 5 | MR07_103 | MR07_260 | MR13J_04_03_0431^b^ | Yes |
| 2012 | Father | 5 | MR07_170 | MR07_450 | MR13J_07_31_2824^a^ | No |
| 2012 | Father | 5 | MR07_103 | MR07_260 | MR13J_04_05_0525 | Yes |
| 2012 | Father | 5 | MR07_103 | MR07_260 | MR13J_04_05_0572 | Yes |
| 2012 | Father | 5 | MR07_084 | MR07_112 | MR13J_04_06_0600 | Yes |
| 2012 | Father | 5 | MR07_103 | MR07_260 | MR13J_04_24_1414 | Yes |
| 2012 | Father | 5 | MR07_126 | MR07_237 | MR13J_05_19_2520 | Yes |
| 2012 | Father | 5 | MR07_262 | MR07_630 | MR13J_05_20_2583 | Yes |
| 2012 | Mother | 5 | MR07_262 | MR07_577 | MR13J_04_22_1351^b^ | Yes |
| 2012 | Mother | 5 | MR07_126 | MR07_237 | MR13J_05_08_2049^b^ | Yes |
| 2012 | Mother | 5 | MR07_427 | MR07_237 | MR13J_05_08_2049^b^ | Yes |
| 2012 | Mother | 5 | MR07_169 | MR07_638 | MR13J_03_07_0007 | Yes |
| 2012 | Mother | 5 | MR07_169 | MR07_638 | MR13J_03_07_0009 | Yes |
| 2012 | Mother | 5 | MR07_169 | MR07_638 | MR13J_03_11_0022 | Yes |
| 2012 | Mother | 5 | MR07_169 | MR07_638 | MR13J_03_13_0040 | Yes |
| 2012 | Mother | 5 | MR07_199 | MR07_122 | MR13J_03_30_0227 | Yes |
| 2012 | Mother | 5 | MR07_173 | MR07_514 | MR13J_04_01_0272 | Yes |
| 2012 | Mother | 5 | MR07_191 | MR07_156 | MR13J_04_01_0284 | Yes |
| 2012 | Mother | 5 | MR07_248 | MR07_078 | MR13J_04_01_0288 | Yes |
| 2012 | Mother | 5 | MR07_262 | MR07_577 | MR13J_04_01_0300 | Yes |
| 2012 | Mother | 5 | MR07_209 | MR07_190 | MR13J_04_02_0399 | Yes |
| 2012 | Mother | 5 | MR07_169 | MR07_638 | MR13J_04_09_0670 | Yes |
| 2012 | Mother | 5 | MR07_123 | MR07_444 | MR13J_04_11_0731 | Yes |
| 2012 | Mother | 5 | MR07_209 | MR07_190 | MR13J_04_14_0885 | Yes |
| 2012 | Mother | 5 | MR07_169 | MR07_638 | MR13J_04_14_0908 | Yes |
| 2012 | Mother | 5 | MR07_262 | MR07_577 | MR13J_04_14_0945 | Yes |
| 2012 | Mother | 5 | MR07_209 | MR07_190 | MR13J_04_15_0949 | Yes |
| 2012 | Mother | 5 | MR07_209 | MR07_190 | MR13J_04_17_1048 | Yes |
| 2012 | Mother | 5 | MR07_209 | MR07_190 | MR13J_04_17_1081 | Yes |
| 2012 | Mother | 5 | MR07_209 | MR07_190 | MR13J_04_18_1106 | Yes |
| 2012 | Mother | 5 | MR07_209 | MR07_190 | MR13J_04_18_1135 | Yes |
| 2012 | Mother | 5 | MR07_262 | MR07_577 | MR13J_04_20_1152 | Yes |
| 2012 | Mother | 5 | MR07_262 | MR07_577 | MR13J_04_20_1243 | Yes |
| 2012 | Mother | 5 | MR07_123 | MR07_444 | MR13J_04_22_1368 | Yes |
| 2012 | Mother | 5 | MR07_123 | MR07_444 | MR13J_04_24_1402 | Yes |
| 2012 | Mother | 5 | MR07_173 | MR07_514 | MR13J_04_24_1456 | Yes |
| 2012 | Mother | 5 | MR07_745 | MR07_525 | MR13J_04_27_1561 | No |
| 2012 | Mother | 5 | MR07_745 | MR07_525 | MR13J_04_27_1572 | Yes |
| 2012 | Mother | 5 | MR07_123 | MR07_444 | MR13J_04_27_1590 | Yes |
| 2012 | Mother | 5 | MR07_169 | MR07_638 | MR13J_04_27_1605 | Yes |
| 2012 | Mother | 5 | MR07_191 | MR07_156 | MR13J_04_27_1610 | Yes |
| 2012 | Mother | 5 | MR07_191 | MR07_156 | MR13J_04_27_1624 | Yes |
| 2012 | Mother | 5 | MR07_262 | MR07_577 | MR13J_04_27_1631 | Yes |
| 2012 | Mother | 5 | MR07_262 | MR07_577 | MR13J_04_29_1654 | Yes |
| 2012 | Mother | 5 | MR07_429 | MR07_219 | MR13J_04_29_1687 | Yes |
| 2012 | Mother | 5 | MR07_123 | MR07_444 | MR13J_04_29_1736 | Yes |
| 2012 | Mother | 5 | MR07_169 | MR07_638 | MR13J_04_29_1753 | Yes |
| 2012 | Mother | 5 | MR07_191 | MR07_156 | MR13J_05_03_1861 | Yes |
| 2012 | Mother | 5 | MR07_057 | MR07_443 | MR13J_05_03_1893 | Yes |
| 2012 | Mother | 5 | MR07_191 | MR07_156 | MR13J_05_06_1998 | Yes |
| 2012 | Mother | 5 | MR07_191 | MR07_156 | MR13J_05_06_2017 | Yes |
| 2012 | Mother | 5 | MR07_126 | MR07_237 | MR13J_05_08_2033 | Yes |
| 2012 | Mother | 5 | MR07_126 | MR07_237 | MR13J_05_08_2042 | Yes |
| 2012 | Mother | 5 | MR07_191 | MR07_156 | MR13J_05_22_2590 | Yes |
| 2012 | Mother | 5 | MR07_126 | MR07_237 | MR13J_08_07_2829 | Yes |
| 2012 | Mother | 5 | MR07_162 | MR07_204 | MR13J_08_10_2831 | Yes |
| 2012 | Mother | 5 | MR07_126 | MR07_237 | MR13J_08_26_2847 | Yes |
| 2013 | Mother | 5 | MR08_329 | MR08_619 | MR14J_04_23_030 | Yes |
| 2013 | Mother | 5 | MR08_329 | MR08_619 | MR14J_04_26_028 | Yes |

^a^ Issues related genotyping the grandparents and the grandoffspring at an additional four loci resulted in the assignment’s exclusion from subsequent analyses.

^b^ Grandoffspring that were assigned to two or three grandparent pairs based on 11 microsatellite loci were excluded from all analyses.

^c^ Changed classification from Mother to Father following identification of genotyping errors made using the sex-linked marker *Oty3*.
